# Integrating bioinformatics and multiple machine learning to identify mitophagy-related targets for the diagnosis and treatment of diabetic foot ulcers: evidence from transcriptome analysis and drug docking

**DOI:** 10.3389/fmolb.2024.1420136

**Published:** 2024-07-09

**Authors:** Hui Guo, Kui Xiao, Yanhua Zheng, Jianchun Zong

**Affiliations:** ^1^ Department of Emergency, The Second Affiliated Hospital, Chongqing Medical University, Chongqing, China; ^2^ Department of Plastic Surgery, Guangzhou Red Cross Hospital, Jinan University, Guangzhou, China; ^3^ Department of Critical Medicine, Wusong Hospital, Zhongshan Hospital, Fudan University, Shanghai, China

**Keywords:** diabetic foot ulcers, mitophagy-related genes, machine learning algorithms, artificial neural network, molecular docking method

## Abstract

**Background:**

Diabetic foot ulcers are the most common and serious complication of diabetes mellitus, the high morbidity, mortality, and disability of which greatly diminish the quality of life of patients and impose a heavy socioeconomic burden. Thus, it is urgent to identify potential biomarkers and targeted drugs for diabetic foot ulcers.

**Methods:**

In this study, we downloaded datasets related to diabetic foot ulcers from gene expression omnibus. Dysregulation of mitophagy-related genes was identified by differential analysis and weighted gene co-expression network analysis. Multiple machine algorithms were utilized to identify hub mitophagy-related genes, and a novel artificial neural network model for assisting in the diagnosis of diabetic foot ulcers was constructed based on their transcriptome expression patterns. Finally, potential drugs that can target hub mitophagy-related genes were identified using the Enrichr platform and molecular docking methods.

**Results:**

In this study, we identified 702 differentially expressed genes related to diabetic foot ulcers, and enrichment analysis showed that these genes were associated with mitochondria and energy metabolism. Subsequently, we identified hexokinase-2, small ribosomal subunit protein us3, and l-lactate dehydrogenase A chain as hub mitophagy-related genes of diabetic foot ulcers using multiple machine learning algorithms and validated their diagnostic performance in a validation cohort independent of the present study (The areas under roc curve of hexokinase-2, small ribosomal subunit protein us3, and l-lactate dehydrogenase A chain are 0.671, 0.870, and 0.739, respectively). Next, we constructed a novel artificial neural network model for the molecular diagnosis of diabetic foot ulcers, and the diagnostic performance of the training cohort and validation cohort was good, with areas under roc curve of 0.924 and 0.840, respectively. Finally, we identified retinoic acid and estradiol as promising anti-diabetic foot ulcers by targeting hexokinase-2 (−6.6 and −7.2 kcal/mol), small ribosomal subunit protein us3 (−7.5 and −8.3 kcal/mol), and l-lactate dehydrogenase A chain (−7.6 and −8.5 kcal/mol).

**Conclusion:**

The present study identified hexokinase-2, small ribosomal subunit protein us3 and l-lactate dehydrogenase A chain, and emphasized their critical roles in the diagnosis and treatment of diabetic foot ulcers through multiple dimensions, providing promising diagnostic biomarkers and targeted drugs for diabetic foot ulcers.

## 1 Introduction

Diabetes mellitus (DM) is a chronic metabolic disease characterized by hyperglycemia (Brody, 2012), and the number of patients with DM continues to increase as the global society ages (Hart et al., 2017). Among the many diabetes-induced complications, the problem of diabetic foot ulcers (DFU) appears to be particularly serious and is widely regarded as one of the most challenging (GBD, 2019 Diseases and Injuries Collaborators, 2020). Recent studies have shown that diabetic foot ulcers are the main contributor to non-traumatic lower limb amputations, with diabetic foot ulcers leading to 40% to 60% of all non-traumatic amputations (Jude et al., 2001). And diabetic foot accounts for 19%∼34% of diabetic patients, with a high mortality rate of 50%∼70% within 5 years (McDermott et al., 2023), and more than 1/3 of diabetic foot ulcers require amputation surgery (Armstrong et al., 2023). The high morbidity, mortality, and disability of the diabetic foot greatly diminishes patients’ quality of life and imposes a heavy socioeconomic burden (Armstrong et al., 2017). Additionally, DFU is also a complex mixture of neuropathy and infection (Villa et al., 2024). Previous studies have shown that foot infections also increase the risk of lower limb amputation in DM patients (Pitocco et al., 2019; Ruder, 2024; Wei et al., 2024), and its prevalence has risen from 4% to 7% (Pitocco et al., 2019). Diabetic foot infection (DFI) is a pathological condition that is primarily caused by the invasion and multiplication of microorganisms in host tissues resulting in an inflammatory response that usually results in tissue damage (Coye et al., 2024; Senneville et al., 2024; Villa et al., 2024; Wang B. et al., 2024). DFI usually occur after the breakage of the skin’s protective coating, and the most common breakage is the DFU, which involves at least the epidermis and part of the dermis (Senneville et al., 2024). Due to the fact that DFUs are often associated with vasculopathy and recurrent microbial infections, which contributes to high patient morbidity and mortality (Xiong et al., 2020). Thus, the main aim of DFU treatment is to control infection and promote wound healing (Primadhi et al., 2023).

However, the current clinical diagnosis and treatment of DFU also face challenges, with the clinical diagnosis of DFU being overly dependent on the clinical experience of clinicians and lacking effective treatments (Armstrong et al., 2017; Matos et al., 2018; Purwanti et al., 2024; Wang F. et al., 2024; Wang K. et al., 2024). At the present stage, the treatment of DFU patients mainly includes debridement, wound dressing, anti-infection treatment, peripheral vasculopathy treatment, strict glycemic control, and amputation (McIllhatton et al., 2021; Orlando et al., 2021; Ahmed et al., 2022; Slomski, 2022; Jeffcoate et al., 2024), but the therapeutic efficacy of these methods is still unsatisfactory and has not yet achieved a satisfactory clinical results, that is, they do not fully control the underlying metabolic lesions (Bardill et al., 2022; Chen et al., 2024; Yi et al., 2024; Zhang G. et al., 2024; Zhang N. et al., 2024). Thus, it is urgent to further identify novel biomarkers of DFU to improve the current dilemma of clinical diagnosis and treatment of DFU.

Mitochondria are the energy powerhouses of the cell, responsible not only for ATP production, but also for regulating calcium homeostasis, responding to oxidative stress, and modulating cell death processes (Li A. et al., 2022). Through its own specialized autophagic program, mitochondrial autophagy precisely removes destroyed mitochondria, and this mechanism is critical for maintaining mitochondrial functionality and ensuring intracellular homeostasis (Onishi et al., 2021). However, mitochondrial autophagy, an important mitochondrial quality control mechanism, leads to the gradual accumulation of mitochondrial DNA (mtDNA) mutations in response to reactive oxygen species (ROS) stress, as well as a decrease in intracellular mitochondrial membrane potential and depolarization damage, eventually leading to cell death (Lu et al., 2023). Previous studies have shown a strong association between DFU and mitophagy (Qi et al., 2023; Wang et al., 2023). The accumulation of ROS can damage the extracellular matrix and endothelial cells, impede the formulation of new blood vessels, and delay the wound healing process (Li et al., 2018). At the same time, the increase in ROS may also exacerbate the inflammatory response, making it difficult for diabetic foot ulcers to heal (Huang et al., 2020). Therefore, reducing the release of ROS appears to be crucial for improving the healing of DFU. Furthermore, in diabetic patients, a persistent hyperglycemic state may trigger impairment of mitochondrial function, which in turn exacerbates the cellular stress response and promotes cell death (Wronka et al., 2022). Enhanced oxidative stress and weakened protective mechanisms in diabetic patients can lead to the accumulation of ROS (Rendra et al., 2019). In this context, mitophagy activators present potential as novel therapeutic approaches for diabetes (Shan et al., 2022). By eliminating dysfunctional mitochondria, mitochondrial autophagy contributes to the reduction of ROS production and the alleviation of oxidative stress (Rovira-Llopis et al., 2017; Zhang et al., 2019), which is particularly important for the healing of DFU. Therefore, it is necessary to develop mitophagy-related genes for the diagnosis and treatment of DFU through whole transcriptome analysis.

In recent decades, machine learning has evolved from a peripheral technology to become a cornerstone of healthcare data analytics (Haug and Drazen, 2023; Shibue, 2023), which is highly effective in identifying disease-relevant biomarkers, making accurate clinical diagnoses, and determining optimal therapeutic regimens in the field of biomedicine (Le et al., 2021). Due to advances in high-throughput sequencing technology and the integration of machine learning in the medical field, many new methods for identifying molecular targets for various diseases are now available (Wu et al., 2023). Clinicians can now utilize biomarkers for early diagnosis of DFU to initiate timely interventions (Wang et al., 2021; Li Y. et al., 2022; Shi et al., 2023; Shi et al., 2024). It has been previously shown that the integration of bioinformatics and machine learning has become an important tool for identifying novel biomarkers associated with DFU (Khandakar et al., 2021; Chemello et al., 2022; Guan et al., 2024; Hettinger et al., 2024).

In this study, we systematically investigated the changes in the expression patterns of mitophagy-related genes in DFU based on DFU whole transcriptome expression data. Immediately after that, we found that hexokinase-2 (HK2), small ribosomal subunit protein us3 (RPS3), and l-lactate dehydrogenase A chain (LDHA) can be indicated using multiple bioinformatic algorithms, that can be used as hub mitophagy-related genes of DFU (DMGs) in DFU. Meanwhile, we validated the importance of DMGs in DFU through multiple dimensions. In conclusion, the present study takes mitophagy as the starting point, and through the combination of multiple bioinformatic analysis techniques and machine learning algorithms to identify novel targets that can be used for the diagnosis and treatment of DFU, which will provide promising targets for the clinical diagnosis and treatment of DFU.

## 2 Materials and methods

### 2.1 Data downloading and processing

In this study, the bulk RNA-seq datasets relevant to DFU were acquired from the gene expression omnibus (GEO) (https://www.ncbi.nlm.nih.gov/geo/), with Table 1 displaying the fundamental information of these datasets. The training cohort comprised 33 samples from GSE134431 and GSE80178, while the validation cohort included 32 samples from GSE7014 and GSE68183. “ComBat” from the R package “sva” was employed to remove batch effects within both the training and validation cohorts (Leek et al., 2012). The effectiveness of this de-batching effect was assessed through principal component analysis (PCA).

**TABLE 1:** The basic information regarding the dataset in the present study.

Cohort division	Dataset source	Annotation platform	Ctrl samples	DFU samples
Training cohort	GSE80178	GPL16686	3	9
	GSE134431	GPL18573	8	13
Validation cohort	GSE68183	GPL16686	3	3
	GSE7014	GPL570	6	20

### 2.2 Gene expression difference analysis

In this study, we compared the whole transcriptome expression data of DFUs and Ctrls using the R package “limma” to identify differentially expressed genes (DEGs) in both clusters at a P-value of < 0.05. P-values were computed using the Wilcoxon rank-sum test.

### 2.3 Weighted gene co-expression network analysis

We used the “WGCNA” R package to identify DEGs associated with DFUs (Langfelder and Horvath, 2008). We first determined the appropriate soft threshold (β) and transformed the expression data matrix into a topological overlap matrix (TOM). Based on the TOM difference metric, we set the minimum module size to 200 and compute the module eigengenes (ME). Subsequently, gene significance (GS) and module significance (MS) were calculated by correlating the identified modules with the phenotypic data of the DFUs, which helped to analyze the correlation between each module and the DFUs.

### 2.4 Downloading of mitophagy-related genes

The mitophagy-related genes (MRGs) used in this study were obtained from the GeneCards database (https://www.genecards.org/). Specifically, we firstly entered the GeneCards database homepage, entered “mitophagy” as the keyword in the search panel, selected the gene type as “protein coding.” The score was set to greater than 2.5, resulting in a total of 417 MRGs (Supplementary Table S1).

### 2.5 Functional enrichment analysis

In order to identify the primary biological processes and functions the 22 DFU-related MRGs were enriched, Gene Ontology (GO) and Kyoto Encyclopedia of Genes and Genomes (KEGG) analyses were conducted utilizing the R package “clusterProfler” (Yu et al., 2012).

### 2.6 Identification of DMGs using machine learning algorithms

We utilized three machine learning algorithms to identify DMGs: support vector machine recursive feature elimination (SVM-RFE), least absolute shrinkage and selection operator (LASSO), and random forest (RF). We applied the RF algorithm from the “randomForest” package, the LASSO algorithm from the “glmnet” package, and the SVM-RFE algorithm from the “e1071” package for screening the 22 DFU-related MRGs to identify potential candidate genes (Noble, 2006; Vasquez et al., 2016; Paul et al., 2018).

### 2.7 Evaluation of the diagnostic performance of DMGs for DFU

We utilized the R software package “pROC” to further evaluate the diagnostic potential of the identified DMGs for DFU. Using the “roc” function of the “pROC” package, we conducted receiver operating characteristic (ROC) analyses. The final area under the ROC curve (AUC) results were calculated using the “ci” function of “pROC.”

### 2.8 Construction of the ANN model

We used the R package “neuralnet” to develope an ANN model. Two data sets, GSE134431 and GSE80178, were used as the training set of the ANN, and two data sets, GSE7014 and GSE68183, were used as the validation set of the ANN. The resulting ANN model featured three main layers: an input layer, a hidden layer, and an output layer.

### 2.9 Gene set enrichment analysis

Based on the median value of the DMGs expression, we clustered the DFU cohort and conducted gene set enrichment analysis (GSEA) to ascertain the correlation between these genes and biological functions.

### 2.10 Immune infiltration analysis

We assessed immune cell infiltration in DFU patients using the R packages “GSVA” and “GSEABase” (Hänzelmann et al., 2013). First, genetic information of 28 immune cell genomes (Supplementary Table S2) was obtained from the TISIDB database (http://cis.hku.hk/TISIDB/). Subsequently, ssGSEA scores were calculated for each sample based on these 28 immune cell genomes. The infiltration levels of different immune cells were visualized using the “pheatmap” R package.

### 2.11 Molecular docking

We utilized the Enrichr platform (https://maayanlab.cloud/Enrichr/) to identify drugs targeting DMGs. The molecular structures of the drugs were sourced from the PubChem database (https://pubchem.ncbi.nlm.nih.gov/). Simultaneously, we retrieved the 3D structures of HK2 (PDB ID: 6Q6N; resolution: 1.63 Å), RPS3 (PDB ID: 4ZVV; resolution: 2.20 Å), and LDHA (PDB ID: 5W8J; resolution: 1.55 Å) from the PDB (https://www.rcsb.org/). Protein and drug files were converted to PDBQT format, excluding water molecules but including polar hydrogen atoms. Using AutoDock tools, we prepared the ligand and protein files, conducted docking simulations, and visualized the interactions with PyMOL.

### 2.12 Statistical analysis

In this study, we performed all statistical analyses of bioinformatics in R language. The analysis of variance (ANOVA) method was employed to statistically analyze multi-group data, while the wilcoxon rank sum test was used to compare two groups. Statistical significance was defined as P < 0.05 for all statistical analyses.

## 3 Results

### 3.1 Differential analysis of gene expression

**FIGURE 1:**
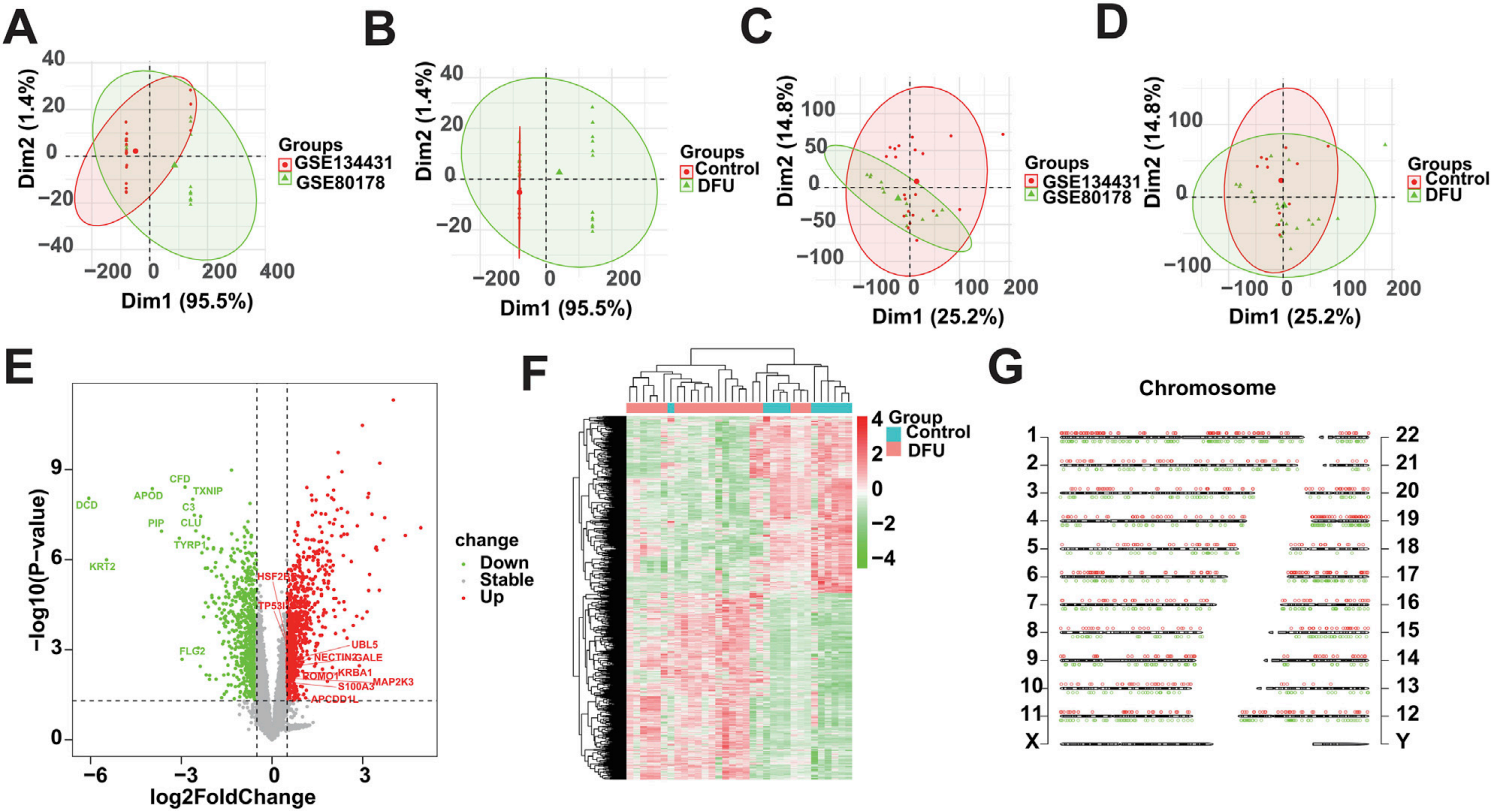
Identification of DEGs based on bulk RNA-seq. **(A,B)** Distributional characteristics of the unprocessed data. **(C,D)** Distribution characteristics of the processed data. **(E)** Volcano plot showing the DEGs between DFUs and Controls. **(F)** Heatmap showing the overall landscape of the DEGs between DFUs and Controls. **(G)** Chromosome map showing the distribution of DEGs on chromosomes.

In this study, we used the 33 samples contained in GSE134431 and GSE80178 as the training cohort, but due to the batch effect between different datasets, we need to remove the batch effect caused by the merging of GSE134431 and GSE80178 in the pre-processing of the data, to make the data in the same dimension and comparable. The data pattern of the training cohort before merging is shown in Figures 1A, B, which shows that the data are not in the same dimension in multiple aspects. After removing the batch effect, the data pattern is shown in Figures 1C, D. The results show that the samples are independent of each other and evenly dispersed, meaning that the data are in the same dimension and comparable, and can be included in subsequent analyses. Next, we used the corrected matrix for differential gene expression analysis (Figure 1E), resulting in a total of 1,665 DEGs, of which 854 were upregulated genes and 811 were down-regulated genes (Supplementary Table S3). The whole expression landscape of these DEGs was shown in Figure 1F, that is, their expression was significantly different between the control and DFU group. Finally, we visualized these 1,665 DEGs on chromosomes and showed that they were evenly distributed on chromosomes and underwent deregulated expression (Figure 1G).

### 3.2 Identification of DFU-related DEGs

**FIGURE 2:**
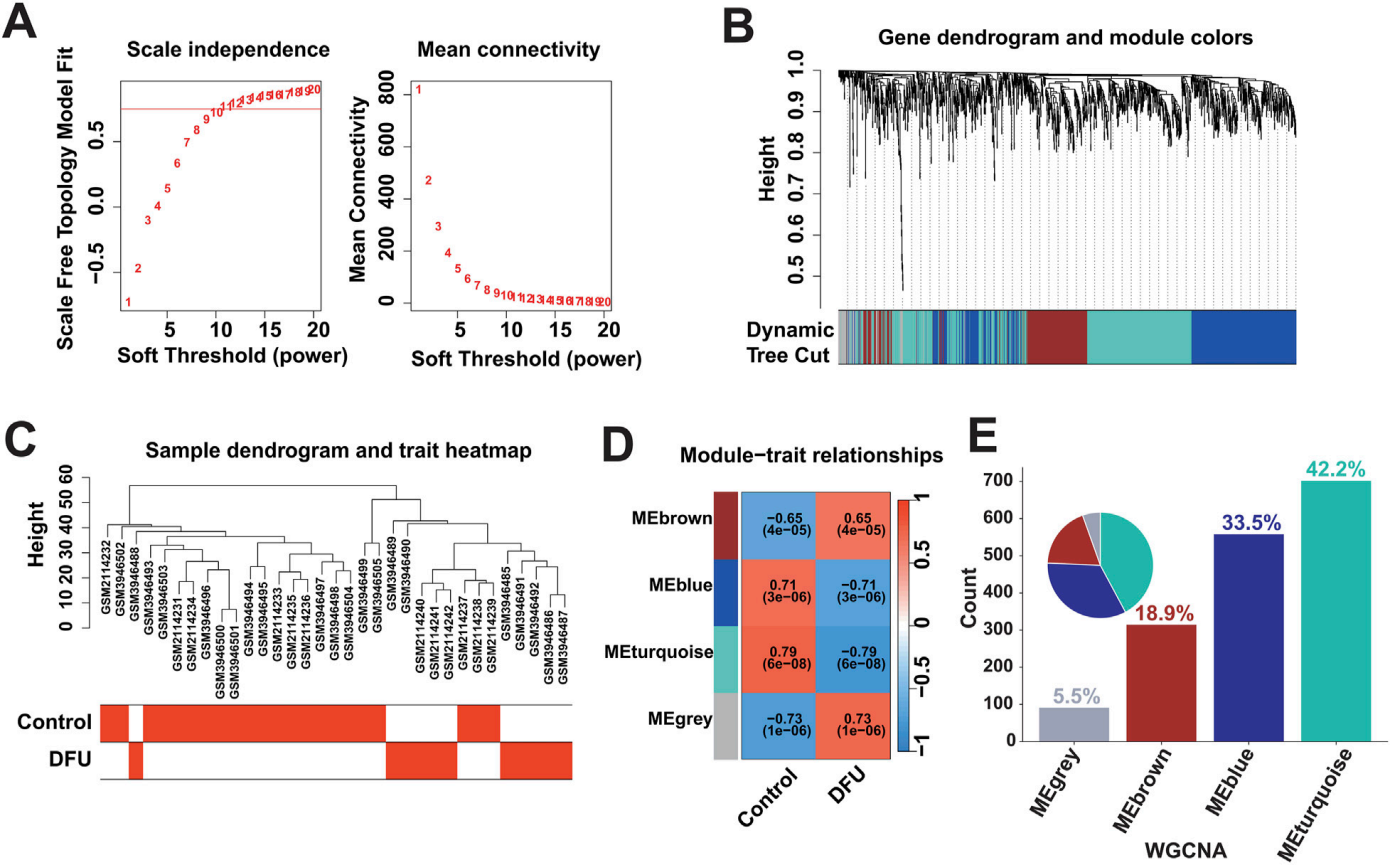
Identification of DFU-related DEGs based on bulk RNA-seq. **(A)** Construction of the unscaled co-expression network. **(B)** Merging of similar modules. **(C)** Sample dendrogram and trait heatmap. **(D)** The correlation between modules and disease phenotypes. **(E)** Statistics on the proportion of the number of module genes.

Dysregulated expression of genes may be caused by multiple factors, that is, to identify DFU-related DEGs, we further performed weighted gene co-expression network analysis. Based on the optimal soft threshold power β = 10 (unscaled R2 = 0.9), the genes were divided into four independent co-expression modules (Figures 2A, B). The clustering dendrogram depicted the sample grouping, with DFU samples being distinctly separated from the control samples (Figure 2C). The correlogram of module-trait relationships highlighted that the turquoise module showed the strongest correlation with DFU (Figure 2D). The turquoise module contained a total of 702 DEGs and had the highest gene percentage (Supplementary Table S4), that is, a total of 42.2% of the dysregulated expression of the DEGs identified in the results mentioned above were related to DFU (Figure 2E), and were thus included in the subsequent analysis as DFU-related DEGs.

### 3.3 The expression pattern of MRGs in DFU altered

**FIGURE 3:**
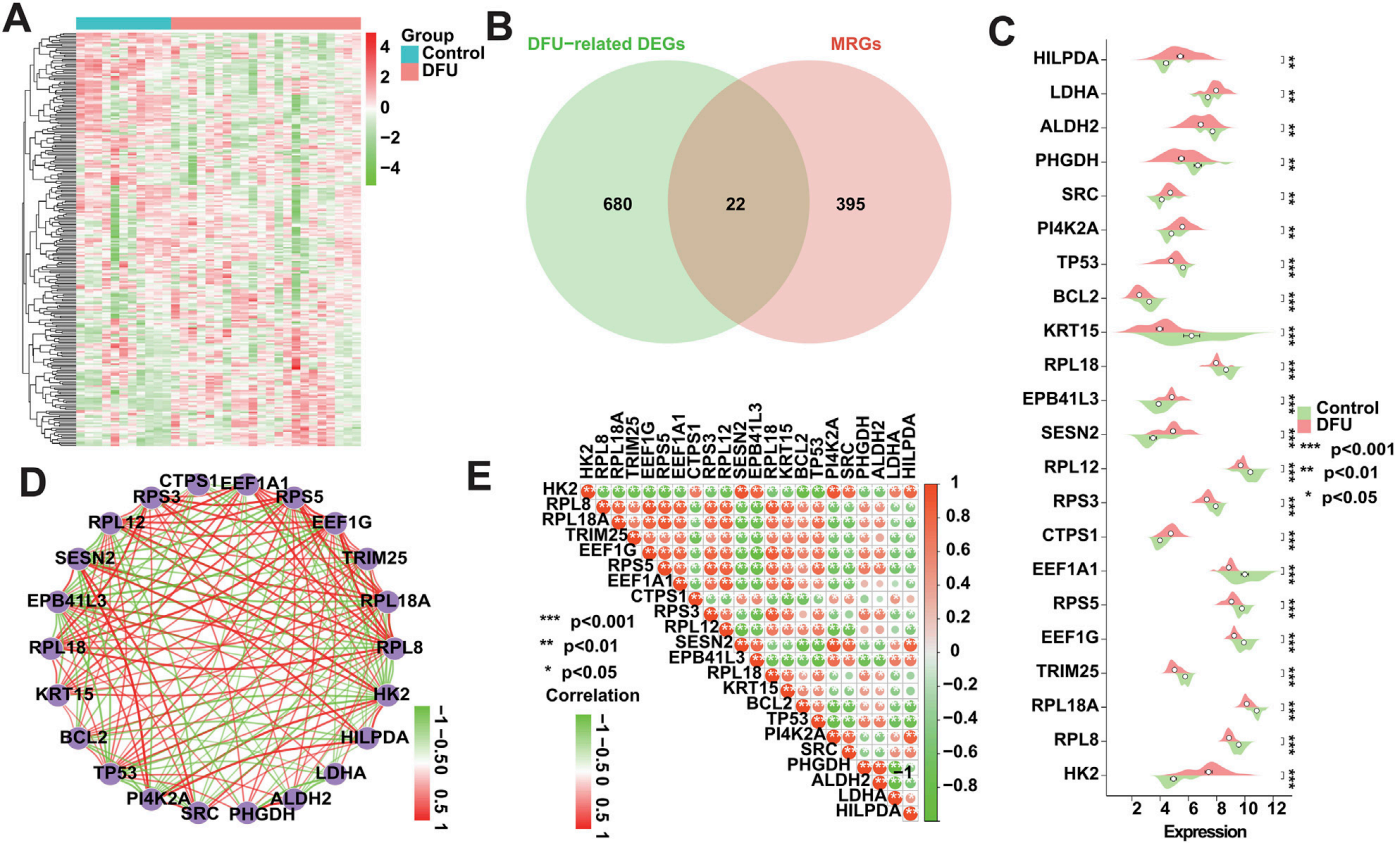
Expression patterns of MRGs in DFU patients. **(A)** Heatmap showing the overall expression landscape of the 417 MRGs. **(B)** Venn plots showing the overlapped genes between 417 MRGs and 702 DFU-related DEGs. **(C)** Box plot showing the expression levels of DFU-related MRGs. **(D)** Gene relationship network diagram of DFU-related MRGs. **(E)** Correlation analysis of DFU-related MRGs. Red and green colors represent positive and negative correlations, respectively. The correlation coefficient was expressed as the area of the pie chart (*p < 0.05, **p < 0.01, ***p < 0.001).

To investigate whether the expression patterns of MRGs in DFUs were altered, we obtained 417 MRGs from the GeneCards database (https://www.genecards.org/) for subsequent studies (Figure 3A). We intersected these 417 MRGs with the 702 DFU-related DEGs identified in the analysis mentioned above, resulting in a total of 22 DFU-related MRGs (Figure 3B), implying that the deregulated expression of these genes affects the onset and progression of DFUs to a certain extent in the form of mitophagy. Immediately after that, we further validated the dysregulated expression of these genes, and the box plot showed that there was a significant difference in their expression between control and DFU groups (Figure 3C). However, genes are not independent of each other, and there would be a certain relationship between their own expression, so we performed correlation analysis on the expression of these 22 DFU-related MRGs, and the results showed that there was a strong expression relationship among them internally (Figures 3D, E). The dysregulated expression of DFU-related MRGs and the synergistic interactions between them suggest that the expression pattern of MRGs in DFU is altered resulting in their ability to contribute to the onset and progression of DFU.

### 3.4 Functional enrichment analysis

**FIGURE 4:**
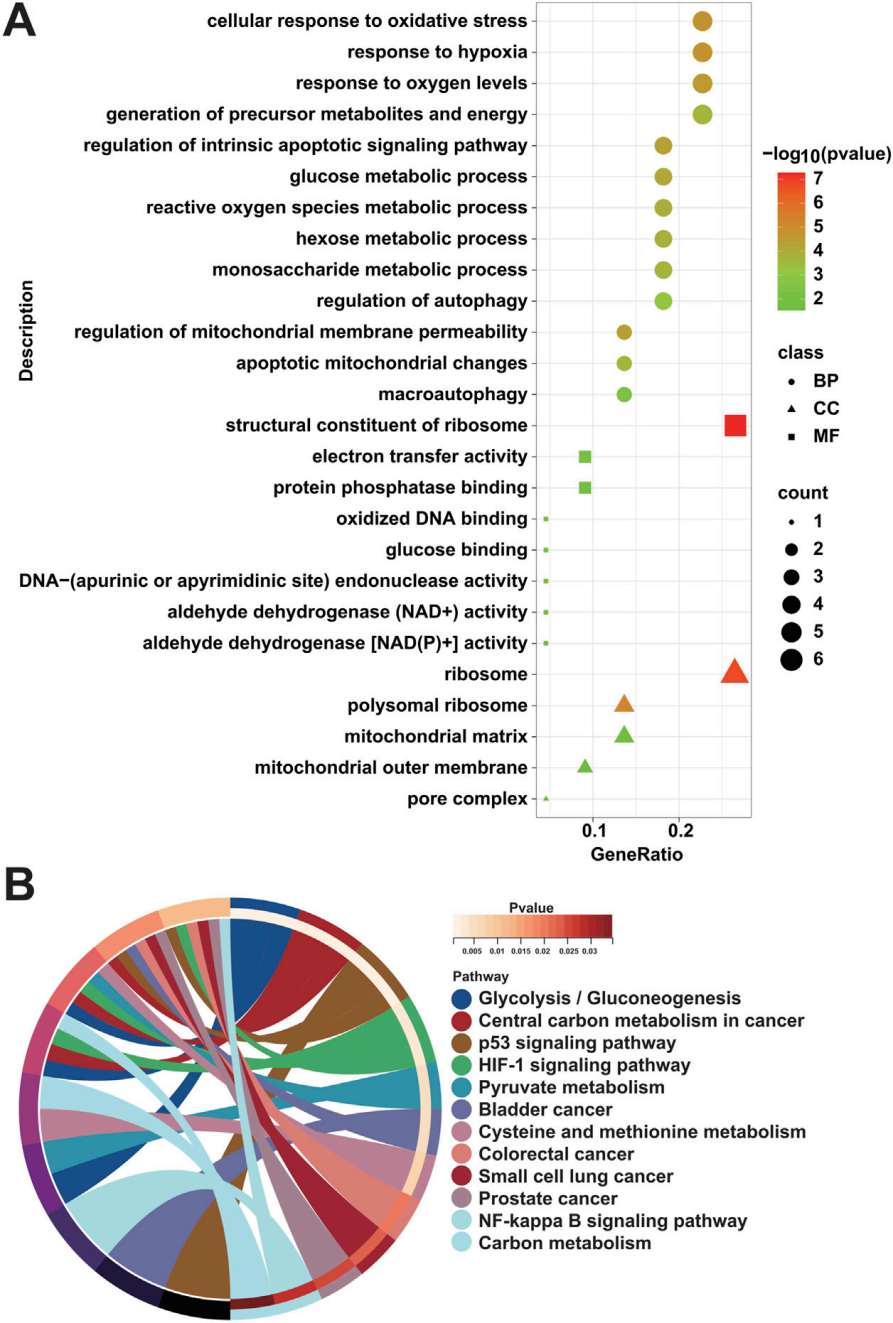
Enrichment analysis of DFU-related MRGs. **(A)** GO enrichment analysis results of DFU-related MRGs. **(B)** KEGG enrichment analysis results of DFU-related MRGs.

To ascertain the biological functions and signaling pathways in which MRGs are mainly enriched to contribute to the progression of DFU, we performed enrichment analysis on the 22 DFU-related MRGs mentioned above. Among them, GO results showed that they were mainly related to mitochondria/autophagy and energy generation and metabolism, such as “mitochondrial outer membrane,” “mitochondrial matrix,” “apoptotic mitochondrial changes,” “regulation of mitochondrial membrane permeability,” “macroautophagy,” “regulation of autophagy,” “aldehyde dehydrogenase [NAD(P)+] activity,” “aldehyde dehydrogenase (NAD+) activity,” “protein phosphatase binding,” “electron transfer activity,” “monosaccharide metabolic process,” “hexose metabolic process,” “reactive oxygen species metabolic process,” “glucose metabolic process,” “generation of precursor metabolites and energy,” “response to oxygen levels,” and “response to hypoxia” (Figure 4A). Additionally, KEGG results were similar to GO results, showing that they were associated with energy generation, amino acid metabolism, and cancer, that is, “Glycolysis/Gluconeogenesis,” “Pyruvate metabolism,” “Cysteine and methionine metabolism,” “Carbon metabolism,” “p53 signaling pathway,” “HIF-1 signaling pathway,” “NF-kappa B signaling pathway,” “Bladder cancer,” “Colorectal cancer,” “Small cell lung cancer,” “Prostate cancer,” and “Central carbon metabolism in cancer” (Figure 4B).

### 3.5 Identification of DMGs using multiple machine learning algorithms

**FIGURE 5:**
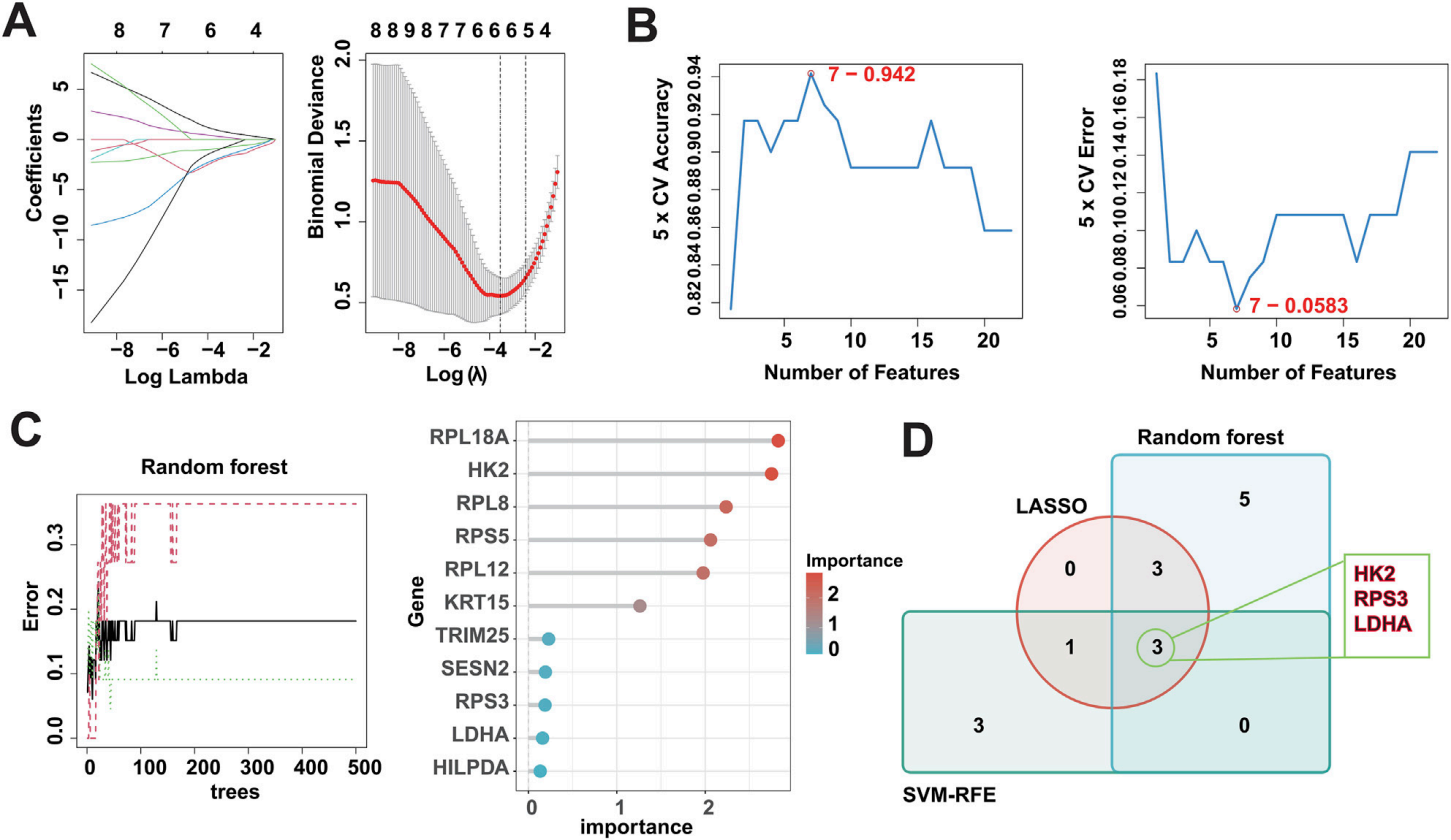
Identification of DMGs using three machine learning algorithms. **(A)** LASSO logistic regression was used to select candidate genes, accompanied by fine-tuning the penalty parameter through a process of tenfold cross-validation. **(B)** The SVM-RFE algorithm was utilized for candidate gene selection. **(C)** The RF algorithm was utilized for candidate gene selection. **(D)** Venn diagram showing the overlapped candidate genes by RF, SVM-RFE, and LASSO algorithms.

In recent years, machine learning has become a major tool for healthcare data analysis and mining, and it is highly effective in identifying disease-related biomarkers and making accurate clinical diagnoses. Clinicians can now utilize biomarkers for early diagnosis of DFU and timely intervention. Thus, the present study analyzed the 22 DFU-associated MRGs mentioned above using multiple machine learning algorithms (Supplementary Table S5). First, we identified 7 candidate genes using the LASSO algorithm (Figure 5A); 7 candidate genes using the SVM-RFE algorithm (Figure 5B); and 11 candidate genes using the RF algorithm (Figure 5C). Subsequently, we crossed the identified candidate genes (Figure 5D), and found that HK2, RPS3, and LDHA could serve as DMGs (Table 2).

**TABLE 2:** Scanning of candidate machines by three machine learning algorithms.

Algorithms	Genes
Lasso	HK2; RPL8; RPL18A; TRIM25; CTPS1; RPS3; LDHA
RandomForest	RPL18A; HK2; RPL8; RPS5; RPL12; KRT15; TRIM25; SESN2; RPS3; LDHA; HILPDA
SVM-REF	HK2; EEF1A1; RPS3; CTPS1; SRC; TP53; LDHA

### 3.6 Diagnostic performance of DMGs

**FIGURE 6:**
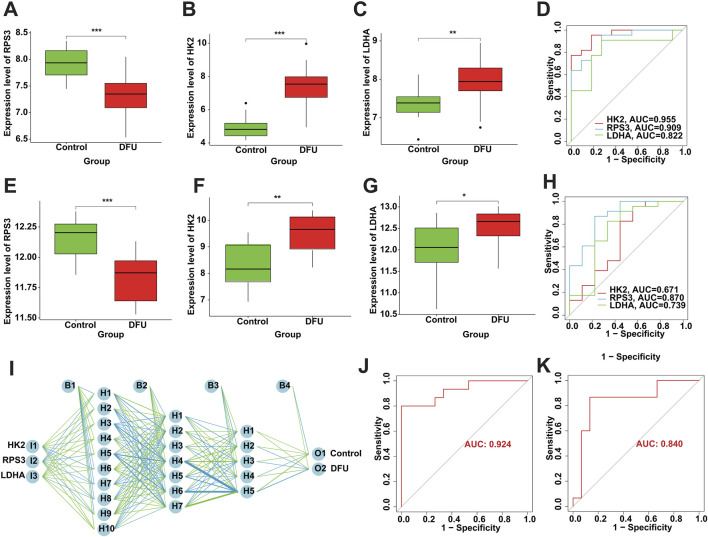
Diagnostic performance of DMGs in DFU. **(A–C)** The expression of the DMGs in the training cohort. **(D)** The ROC of the DMGs in the training cohort. **(E–G)** The expression of the DMGs in the validation cohort. **(H)** The ROC of the DMGs in the validation cohort. **(I)** Construction of ANN based on the DMGs. **(J)** The AUC of the training set with a value of 0.924. **(K)** The AUC of the validation set with a value of 0.840.

The screened DMGs were significantly differentially expressed, suggesting that DMGs may play a potential role in contributing DFU (Figures 6A–C). Furthermore, AUC of DMGs was 0.955 of HK2, 0.909 of RPS3, and 0.822 of LDHA, respectively (Figure 6D). The results of the validation cohort showed that DMGs were also dysregulated, where RPS3 was down-regulated in DFU and HK2 and LDHA were both up-regulated in DFU (Figures 6E–G). DMGs also showed good performance in DFU in the validation set, with AUCs of 0.671 for HK2, 0.870 for RPS3 and 0.739 for LDHA (Figure 6H). Additionally, we also constructed an artificial neural network (ANN) model based on the DMGs. Two data sets, GSE134431 and GSE80178, were used as the training set of the ANN, and two data sets, GSE7014 and GSE68183, were used as the validation set of the ANN. The resulting ANN model featured three main layers: an input layer, a hidden layer, and an output layer (Figure 6I). Table 3 presents the results of the ANN prediction. The training set achieved a prediction accuracy of 90.9%, while the validation set yielded 87.5%. Furthermore, the ANN model has an AUC value of 0.924 in the training set (Figure 6J), and 0.840 in the validation set (Figure 6K). Overall, the ANN model constructed based on the transcriptome of DMGs can assist in the prediction of DFU.

**TABLE 3:** ANN diagnosis effect for the training and validation cohort.

		Training set		Validation set	
		Control	DFU	Control	DFU
Prediction	Control	10	2	8	3
	DFU	1	20	1	20
	Accuracy	90.9%		87.5%	
	AUC	0.924		0.840	

### 3.7 GSEA analysis

**FIGURE 7:**
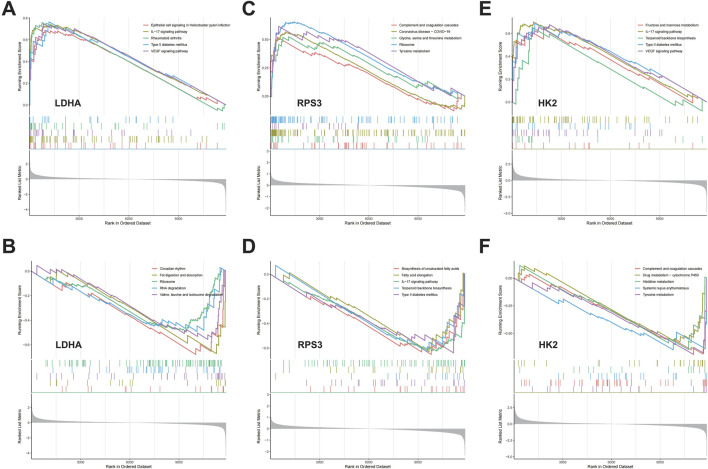
The GSEA of DMGs in DFU. **(A,B)** The GSEA of LDHA in DFU. **(C,D)** The GSEA of RPS3 in DFU. **(E,F)** The GSEA of HK2 in DFU.

We assessed signaling pathways associated with DMGs using GSEA analysis. The results showed that LDHA is enriched in type II diabetes mellitus, rheumatoid arthritis, ribosome, RNA degradation, valine, leucine and isoleucine degradation, fat digestion and absorption, and circadian rhythm (Figures 7A, B). RPS3 is enriched in ribosome, tyrosine metabolism, glycine serine and threonine metabolism, terpenoid backbone biosynthesis, and type II diabetes mellitus (Figures 7C, D). HK2 is enriched in IL-17 signaling pathway, type II diabetes mellitus, terpenoid backbone biosynthesis, systemic lupus erythematosus, and tyrosine metabolism (Figures 7E, F). As mentioned above, DMGs are enriched in diabetes-related biological pathways, such as “Type II diabetes mellitus,” which creates the foundation and necessary conditions for the development of DFU. Additionally, we found that DMGs are also associated with inflammatory signaling pathways and amino acid metabolism signaling pathways, such as “IL-17 signaling pathway,” “Valine, leucine and isoleucine degradation,” “Glycine, serine and threonine metabolism,” “Histidine metabolism,” and “Tyrosine metabolism.” There exists a strong relationship between amino acid metabolism and energy metabolism in organisms (e.g., glycolysis, TCA cycle, respiratory chain, etc.). Amino acids can be metabolized to produce ATP and also provide intermediates for other metabolic pathways to maintain normal life activities. However, dysregulation of amino acid metabolism can also lead to disturbed energy metabolism in organisms, which can activate mitochondrial abnormalities including mitochondrial autophagy, and thus induce inflammatory diseases.

### 3.8 Immune cell infiltration analysis

**FIGURE 8:**
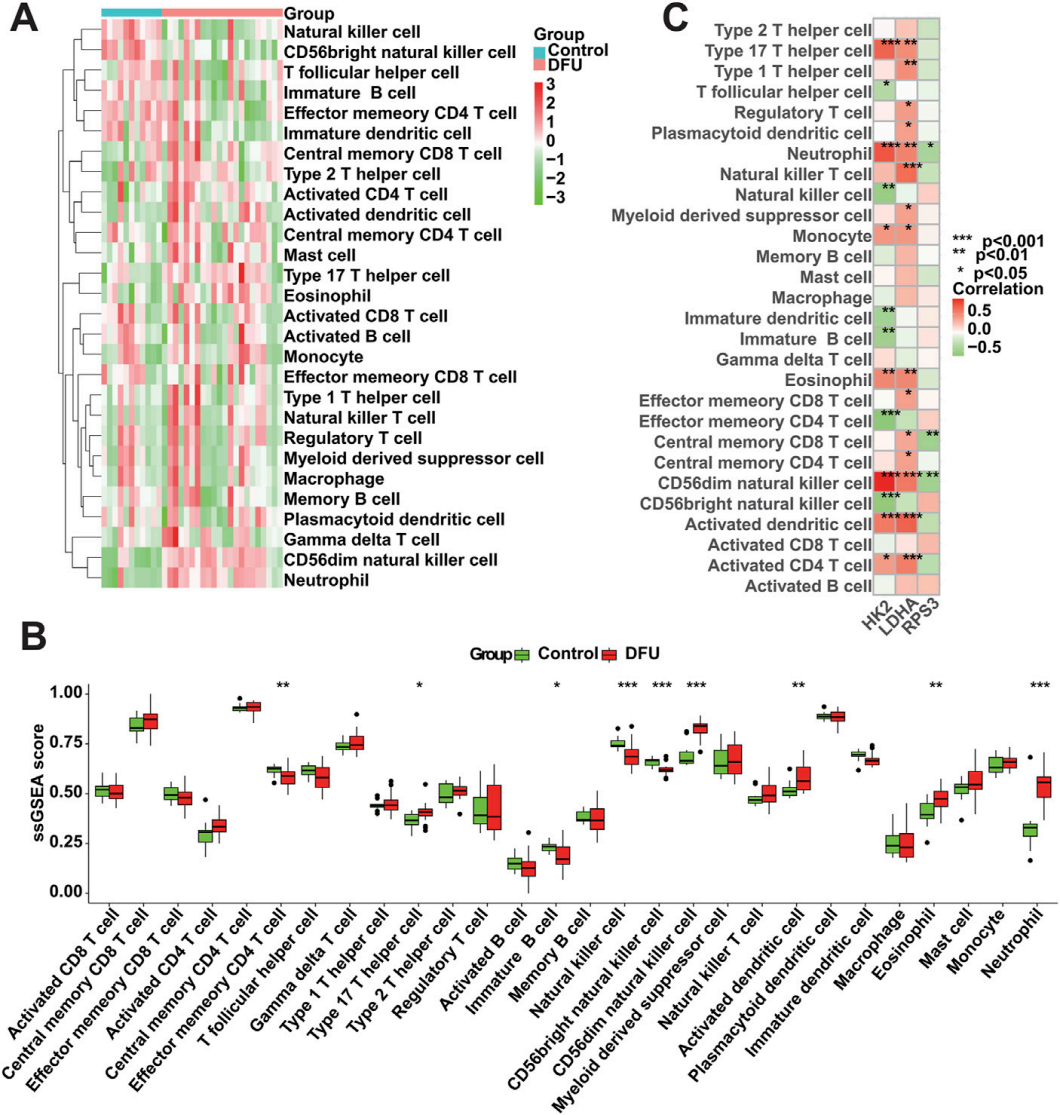
Immune cell infiltration analysis. **(A)** Overall characterization of immune cell abundance. **(B)** The difference of immune cell infiltration abundance between the DFU samples and control samples. **(C)** Correlation between DMGs expression and immune cell infiltration abundance (*p < 0.05, **p < 0.01, ***p < 0.001).

We conducted an assessment of the immunological profile of DFU samples utilizing the ssGSEA algorithm (Supplementary Table S6). Figure 8A showed overall landscape of immune cell infiltration between the DFU and Control groups. Compared with Control samples, DFU samples have higher “Type 17 T helper cell,” “CD56dim natural killer cell,” “Activated dendritic cell” and “Eosinophil” “Neutrophil,” and have lower “Effector memory CD4 T cell,” “Immature B cell,” “Natural killer cell,” “CD56bright natural killer cell” (Figure 8B). Subsequently, we explored the correlation between the expression of DMGs and the aforementioned dysregulated immune cells through correlation analysis. The results showed that there was a significant correlation between the expression of DMGs and the abundance of these immune cells, for example, the expression of HK2 and LDHA was positively correlated with the abundance of Neutrophil, Monocyte, Eosinophil and Activated CD4 T cell, whereas RPS3 expression was negatively correlated with the abundance of Neutrophil and Central memory CD8 T cell (Figure 8C). The significant correlation suggests that the dysregulated expression of DMGs may contribute to the disruption of the immune microenvironment in DFU patients, thereby worsening the inflammatory features that characterize DFU patients.

### 3.9 Molecular docking

**FIGURE 9:**
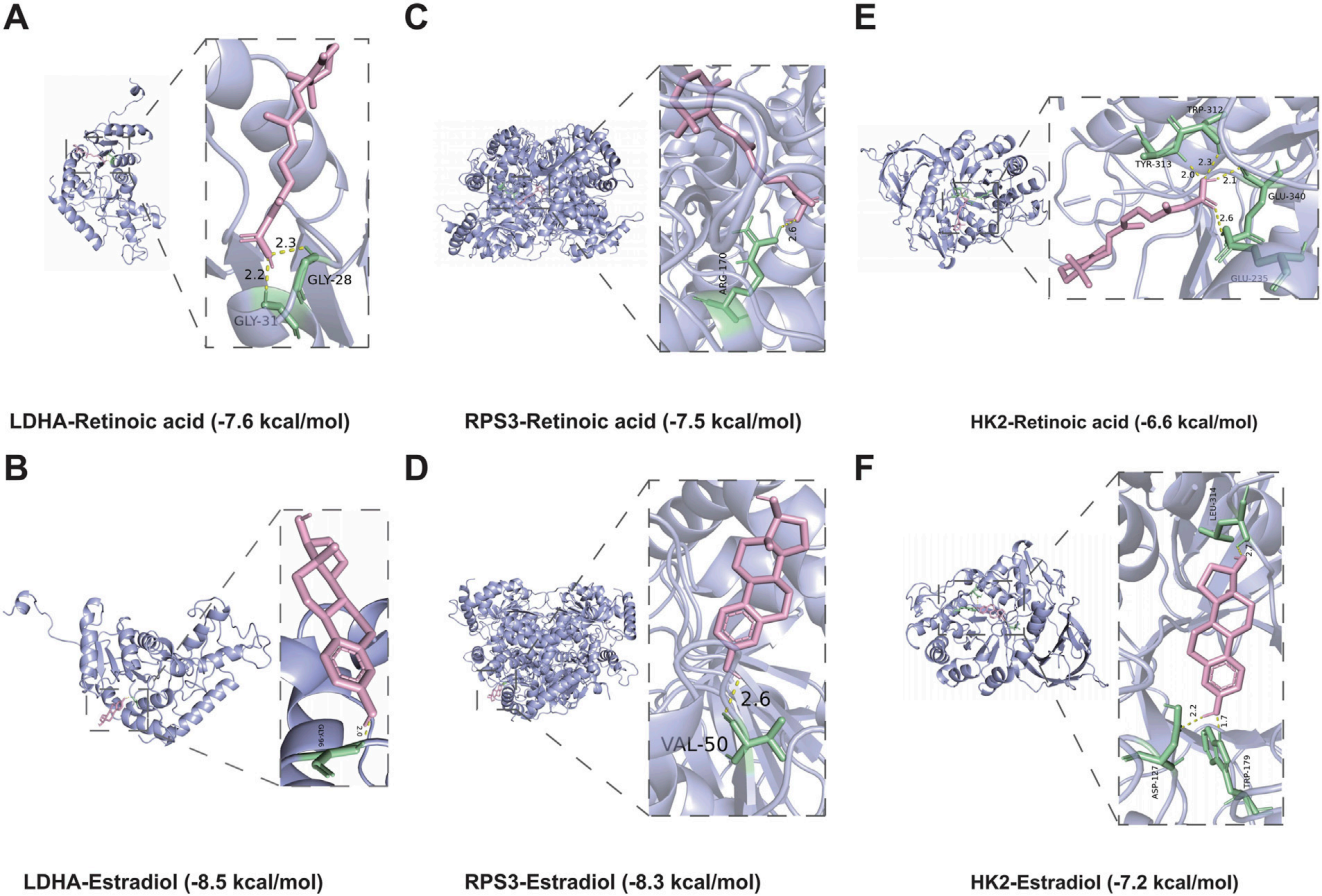
Molecular docking simulations. **(A,B)** The structure of the complex formed by the docking of LDHA with Retinoic acid **(A)**, Estradiol **(B)**. **(C,D)** The structure of the complex formed by the docking of RPS3 with Retinoic acid **(C)**, Estradiol **(D)**. **(E,F)** The structure of the complex formed by the docking of HK2 with Retinoic acid **(E)**, Estradiol **(F)**.

Previous studies has shown that drug discovery typically commences with identifying disease targets, with target-based drug discovery being the predominant approach for developing new drugs. Thus, targeting DMGs may provide a new effective therapeutic option for DFU treatment. Specifically, we identified two drugs capable of targeting DMGs based on the DSigDB database, namely, retinoic acid and estradiol. The results showed that both drugs were able to target DMGs and both had higher binding energies, sequentially LDHA-Retinoic acid (−7.6 kcal/mol) and LDHA-Estradiol (−8.5 kcal/mol) (Figures 9A, B), RPS3-Retinoic acid (−7.5 kcal/mol) and RPS3- Estradiol (−8.3 kcal/mol) (Figures 9C, D), HK2-Retinoic acid (−6.6 kcal/mol), and HK2-Estradiol (−7.2 kcal/mol) (Figures 9E, F). Notably, retinoic acid and estradiol have been shown in previous reports to be used in the treatment of skin-related diseases, for example, retinoic acid and its derivatives have potential for the treatment of severe skin diseases (Szymański et al., 2020). Estradiol promotes the production of extracellular matrix (ECM) (Soldano et al., 2010; Aida-Yasuoka et al., 2013; Baker Frost et al., 2021), which helps to enhance skin elasticity and reduce wrinkles (Rzepecki et al., 2019). These also provide the basis for the use of retinoic acid and estradiol in the treatment or alleviation of DFU symptoms.

## 4 Discussion

DFU is one of the primary causes of disability and death in DM patients. Previous studies have shown a strong relationship between DFU and mitophagy (Qi et al., 2023; Wang et al., 2023), and importantly mitophagy activators present potential as a novel therapeutic approach for diabetes (Shan et al., 2022), that is, through elimination of malfunctioning mitochondria, mitophagy helps to reduce the production of ROS and alleviate oxidative stress (Rovira-Llopis et al., 2017; Zhang et al., 2019), which is particularly important for the healing of DFUs. Therefore, the aim of the present study was to investigate the potential of MRGs as DFU biomarkers thereby contributing to the clinical diagnosis and treatment of DFU.

The current study was based on bulk RNA-seq data from DFU. Specifically, we firstly obtained 1,665 genes that were deregulated in DFU by differential analysis, of which 854 were up-regulated and 811 were down-regulated. Immediately after that, we used weighted gene co-expression network analysis to find that 42.2% of the mentioned above 1,665 DEGs, which are DFU-related DEGs, had their deregulated expression associated with the occurrence of DFU. Meanwhile, in order to investigate the role that mitophagy contributes to the occurrence of DFU, we isolated the MRGs in DFU-related DEGs using the MRGs annotated in the GeneCards database for MRGs with potential regulation in DFU, known as DFU-related MRGs. It is important to notice that the genes themselves do not operate and function independently, and their internal expression also has a strong correlation. The results of correlation analysis showed that almost all of the 22 DFU-related MRGs identified had significant correlation in their internal expression, implying that these genes, as the core genes of mitophagy factors, can largely contribute to the occurrence and development of DFU. Secondly, to verify the reliability of these genes, we enriched and analyzed them, and the results showed that they were significantly correlated with mitophagy, energy generation and metabolism, amino acid metabolism, and cancer, which means that our results have a certain reliability. In order to identify DMGs in DFU, we utilized multiple machine learning, and the results showed that HK2, RPS3 and LDHA could be indicated by all the machine learning algorithms.

It is worth mentioning that HK2, RPS3 and LDHA have been reported in previous studies. For example, Hexokinase, the first key enzyme in the glucose metabolic pathway responsible for the conversion of glucose to glucose 6-phosphate, is encoded by HK2. It was previously shown that HK2 expression is insulin-responsive, and in diabetes mellitus, HK2-associated unscheduled glycolysis may be a key initiator of insulin resistance and the development of vascular complications (Rabbani et al., 2022). It has also been demonstrated that inhibition of HK2 up-regulation can block excessive glycolytic responses, which may result in preservation of mitochondrial function and inhibition of diabetic renal fibrosis (Hasegawa et al., 2023). RPS3 is an essential component of the 40S subunit of eukaryotic ribosomes and is directly involved in ribosome maturation and translation initiation. RPS3 is expressed in human pancreatic islet cells in response to the transfer of cytokines from the cytoplasmic to the nuclear compartment. Furthermore, RPS3 is involved in NF-κB signaling, and a genetic increase in the activity of the NF-κB subunit c-Rel leads to protection against human islet cell death (Mokhtari et al., 2009). Meanwhile, ethanol may affect oxidative stress in β-cells by increasing the expression of the RPS3 gene, thereby decreasing their metabolic activity (Shin et al., 2004). Lactate dehydrogenase (LDH) is a metabolic enzyme that catalyzes the interconversion of pyruvate and lactate. LDH is a tetramer consisting of two subunits: muscular and cardiac, encoded by LDHA and LDHB, respectively (Zhang et al., 2022). LDHA catalyzes the reversible conversion of pyruvate to lactate while oxidizing NADH to NAD in anaerobic glycolysis. The inhibition of LDHA under normal physiological conditions and its inappropriate up-regulation in the diabetic milieu is well documented and is predominantly enriched in human islet α cells (Sanchez et al., 2021). In addition, the pathogenesis of diabetic kidney disease (DKD) is associated with LDHA-mediated lactic acidosis, which leads to mitochondrial abnormalities and renal fibrosis in DKD patients (Walbridge et al., 1987). In summary, the DMGs are associated with energy metabolism abnormalities.

Certainly, the performance of DMGs for DFU still needs both single-gene as well as multi-gene tests, including ROC and ANN (Mandair et al., 2023). ANN, as a form of artificial intelligence, has found extensive application in clinical medicine (Kufel et al., 2023; Mandair et al., 2023; Popovic et al., 2023). However, the current clinical methods for diagnosing DFU have limitations, particularly in terms of molecular-level diagnosis, which is often reliant on the clinical experience (Voelker, 2023). Thus, we constructed the ANN model based on DMGs with high prediction accuracy. The AUC values were all more than 0.8. Overall, the ANN constructed based on the transcriptome of DMGs can assist in the prediction of DFU.

Prior research has shown that drug discovery typically commences with identifying disease targets, with target-based drug discovery being the predominant approach for developing new drugs (Paananen and Fortino, 2020; Trajanoska et al., 2023). We identified two drugs capable of targeting DMGs based on the DSigDB database, namely, retinoic acid and estradiol. Retinoic acid and its derivatives have therapeutic potential for severe skin diseases (Szymański et al., 2020). Estradiol promotes extracellular matrix (ECM) production (Soldano et al., 2010; Aida-Yasuoka et al., 2013; Baker Frost et al., 2021), contributes to skin elasticity and reduces wrinkles (Rzepecki et al., 2019). In addition, the results of molecular docking showed a high binding affinity between retinoic acid and estradiol and DMGs, implying that they may be promising therapeutic agents against DFU.

There are also several limitations of this study that need to be clarified. Firstly, the number of DFU samples in the GEO database that could be incorporated into the present study was small, which also limited our possibility to perform additional cohort analyses and validation. Secondly, the clinical information of DFU samples was incomplete, which led to the lack of clinical phenotyping research on DFU in the present study. Finally, this study still needs more biological and clinical experiments for its subsequent analysis and validation.

## 5 Conclusion

In the present study, we performed a series of bioinformatic analyses based on DFU- related transcriptomics data. Specifically, we identified 702 DFU-related DEGs, including 22 MRGs, and the enrichment analysis results showed that these genes are related to mitochondria and energy metabolism. Immediately following this, we used multiple machine learning algorithms (RF, Lasso and SVM-RFE) to collectively identify HK2, RPS3 and LDHA to serve as DMGs. Meanwhile, we constructed a novel ANN model for DFU diagnosis, and the ROC curves of the model showed good performance. Additionally, the results of ssGSEA showed that DMGs could regulate the immune microenvironment of DFU patients. Finally, we found that retinoic acid and estradiol may be promising drugs against DFU. In conclusion, this study identified DMGs through multiple analytical techniques and verified their importance in DFU through multiple dimensions, providing promising targets for the clinical diagnosis and treatment of DFU.

## Data Availability

The datasets presented in this study can be found in online repositories. The names of the repository/repositories and accession number(s) can be found in the article/Supplementary Material.

## Abbreviations

DM, Diabetes mellitus; DFU, Diabetic foot ulcers; DFI, Diabetic foot infection; MRGs, Mitophagy-related genes; DMGs, Hub mitophagy-related genes of DFU; mtDNA, Mitochondrial DNA; ROS, Reactive oxygen species; PCA, Principal component analysis; DEGs, Differentially expressed genes; TOM, Topological overlap matrix; ME, Module eigengene; GS, Gene significance; MS, Module significance; GO, Gene ontology; KEGG, Kyoto encyclopedia of genes and genomes; SVM-RFE, Support vector machine recursive feature elimination; LASSO, Least absolute shrinkage and selection operator; RF, Random forest; ROC, Receiver operating characteristic; AUC, Area under roc curve; ANN, Artificial neural network; GSEA, Gene set enrichment analysis; ssGSEA, single sample gene set enrichment analysis; HK, Hexokinase; HK2, Hexokinase-2; RPS3, Small ribosomal subunit protein us3; LDHA, L-lactate dehydrogenase A chain; DKD, Diabetic kidney disease; ECM, Extracellular matrix.
